# Author's response

**DOI:** 10.52054/FVVO.14.4.053

**Published:** 2023-01-27

**Authors:** Ertan Saridogan

**Affiliations:** University College London Hospital, Women’s Health Division, London, United Kingdom; University College London, Elizabeth Garrett Anderson Institute for Women’s Health, London, United Kingdom

Sir,

I thank Professor Koninckx and his colleagues for their interest in our video article entitled ‘Endometrial preservation during resection of type II and type II submucosal fibroids’ (Vorona and Saridogan, 2022). We focussed on endometrial preservation in this video article with the intention to reduce intrauterine adhesion formation and loss of functional endometrium. Koninckx et al. raise an interesting point in their Letter to Editor and highlight the absence of any significant information on the impact of hysteroscopic myomectomy on the ‘junctional zone’. They highlight the potential importance of the junctional zone in uterine function, its role in uterine peristalsis, placentation, and possible involvement in the development of dysmenorrhoea and adenomyosis.

The junctional zone was first described as a low-intensity band between the endometrium and myometrium in magnetic resonance imaging (MRI) (Hricak et al., 1983). Similarly, ultrasound finding of junctional zone was later described, although there might be differences between the boundries of the zone in MRI and ultra- sound (Mitchell et al., 1990). In addition, there is no overall consensus on terminology either, some groups prefer the term endometrial-myometrial junction, inner myometrium, endometrial-subendometrial subunit, subendometrial myometrium or stratum subvasculare (Naftalin and Jurkovic, 2009). It appears that there is little, if any, difference between the junctional zone and adjacent outer myometrium histologically; junctional zone consists of compact smooth muscle fibres with little extracellular matrix compared with the myometrium proper (Brown et al., 1991) and/or has lower water content (McCarthy et al., 1989). With this uncertain back- ground, it is thought that the junctional zone has a separate embryological origin (paramesonepric or mullerian duct) than the rest of the myometrium (mesenchymal origin), but they both originate from the embryonic mesoderm. This is plausible from an evolutionary point of view, as the junctional zone which is considered to be part of ‘archimetra’ (old uterus) is present in all vertebrate mammals, whereas the outer layers of the myometrium (neometra) are thought to be acquired later in the evolution process (Leyendecker et al., 2022).

Although it has been suggested that submucosal fibroids originate from the inner myometrium (Brosens et al., 2003), it is likely some fibroids originating from the outer myometrium will eventually become submucosal (Naftalin and Jurkovic, 2009). It is suggested that fibroids originating from the inner myometrium should cause a disruption of its uniform hypoechoic echotexture adjacent to the basal endometrium which is normally clearly visible as a white hyperechoic line, whilst the fibroids originating from the outer myometrium should cause a displacement of the junctional zone without affecting its morphological appearance (Naftalin and Jurkovic, 2009). I agree with Koninckx et al. that currently no information is available to differentiate between these two types of submucosal fibroids on ultrasound.

Considering this background, it is quite likely that hysteroscopic myomectomy might cause some degree of impact on the junctional zone, or inner myometrium. This impact is likely to be negligible with type 0 fibroids, when the tumour is completely within the endometrial cavity, and may increase from type I through to type III fibroids. One could argue that hysteroscopic myomectomy removes the fibroid through a ‘gap’ which has been created between the myometrial bundles by the protruding fibroid, therefore without making any ‘incision’ to the junctional zone. It is quite likely that there is some overlying myometrium between the fibroid and uterine cavity with type III fibroids, and that this may be the case for some type II fibroids. Hence, during hysteroscopic myomectomy a layer of myometrium might be resected during removal of these fibroids, or an incision will need to be made with the approach we described in our technique (Vorona and Saridogan, 2022).

There is however no evidence that this has any clinical significance. In fact, published data on the outcome of removal of submucosal fibroids is quite reassuring; pregnancy rates increase and miscarriage rates decline, both returning to levels seen in women without submucosal fibroids (Pritts et al., 2009). Furthermore, even abdominal myomectomy for large submucosal fibroids with an intramural component seem to have the same effect, returning ongoing pregnancy and implantation rates to the levels seen in women without fibroids (Surrey et al., 2005).

Nevertheless, I agree with Koninckx et al. that we need more research on the impact of hysteroscopic myomectomy on the junctional zone and its healing and explore if our method of ‘incision making’ instead of ‘resection’ has better outcomes. The concept of junctional zone incision to treat dysmenorrhoea or prevent endometriosis is very interesting, but we need some data that indicate its plausibility, before we put it to test in a research setting.
